# Efficacy of bone substitute material in preserving volume when placing a maxillary immediate complete denture: study protocol for the PANORAMIX randomized controlled trial

**DOI:** 10.1186/s13063-016-1380-7

**Published:** 2016-05-20

**Authors:** Christophe Rignon-Bret, Alain Hadida, Alexis Aidan, Thien-Huong Nguyen, Gerard Pasquet, Helene Fron-Chabouis, Claudine Wulfman

**Affiliations:** Albert Chevenier Hospital, Assistance Publique – Hôpitaux de Paris, 40 rue de Mesly, 94000 Créteil, France; Biomaterials Department (URB2i, EA4462), Sorbonne Paris Cité, Faculté de Chirurgie Dentaire, Université Paris Descartes, 75006 Paris, France; Sorbonne Paris Cité, Faculté de Chirurgie Dentaire, Université Paris Descartes, 75006 Paris, France; Cabinet de Radiologie Dentaire Echelle Saint-Honoré, 179, rue Saint-Honoré, 75001 Paris, France; Charles Foix Hospital, Assistance Publique – Hôpitaux de Paris, 7 avenue de la République, 94200 Ivry-sur-Seine, France; Louis Mourier Hospital, Assistance Publique – Hôpitaux de Paris, 178 Rue des Renouillers, 92700 Colombes, France

**Keywords:** Clinical trial, Complete denture, Bone substitute, Socket graft

## Abstract

**Background:**

Bone preservation is an essential issue in the context of last teeth extraction and complete edentulism. The intended treatment, whether a complete denture or an implant placement, is facilitated with a voluminous residual ridge. Bone resorption after multiple extractions has not been as well studied as the bone resorption that occurs after the extraction of a single tooth. Recent advances in bone substitute materials have revived this issue. The purpose of this study is to evaluate the interest in using bone substitute material to fill the socket after last teeth extraction in a maxillary immediate complete denture procedure compared with the conventional protocol without socket filling.

**Methods/design:**

A randomized, controlled, clinical trial was designed. The 34 participants eligible for maxillary immediate complete denture were divided into two groups. Complete dentures were prepared despite persistence of the last anterior teeth. The control group received a conventional treatment including denture placement immediately after extractions. In the experimental group, in addition to the immediate denture placement, a xenograft bone-substitute material (Bio-Oss Collagen®) was placed in the fresh sockets. The primary outcome of the study is to compare mean bone ridge height loss 1 year after maxillary immediate complete denture placement, with or without bone-substitute material, in incisor and canine sockets. The secondary outcomes are to compare the average bone ridge height and width loss for each extraction site. An original quantitative evaluation method using cone beam computed tomography was designed for reproducible measurements, with a radio-opaque denture duplicate. Two independent operators perform the radiologic measurements.

**Discussion:**

The immediate complete denture technique limits bone resorption in multiple extraction situations and thus allows better denture retention and better options for implant placement. To compare the benefit of using any bone socket-filling material, we proposed a quantitative evaluation protocol of resorption in the specific case of the last anterior maxillary teeth extraction with immediate denture placement.

**Trial registration:**

ClinicalTrials.gov, NCT02120053. Registered on 18 April 2014.

## Background

Edentulism, or complete tooth loss, represents an important disability leading to poor nutrition and social disadvantages. Despite projections of declining edentulism, the need for complete denture treatment for elderly patients remains high, even in industrialized countries, due to life expectancy increase [1–3]. Dentures are provided to restore oral function (mastication, speech, and deglutition) and improve general well-being. They are placed on the edentulous ridge after teeth extraction and concomitant ridge resorption. Conventional removable dentures are used worldwide to treat complete edentulism. In addition, implant treatment has developed in the last decades for the additional comfort it provides patients with complete edentulism. Whatever the rehabilitation treatment chosen, a large supporting ridge is an advantage because this enhances denture retention, stability, and support and therefore leads to improved comfort and well-being [4, 5]. Moreover, implant placement may be considered under the best conditions [6–8]. Thus, ridge preservation at the time of tooth extraction is important to maximize denture stability and treatment success.

When the last anterior teeth are compromised in the short term because of tooth decay, loss of tooth structure, or periodontal disease, the immediate complete denture technique consists of denture placement immediately after removal of the last teeth [4, 9]. The advantage is immediate rehabilitation of both aesthetics and function. Thus, the patient does not remain toothless and is not confronted with the disability. This situation also leads to a better acceptance of the denture. This technique, widely described and taught, also aims to reduce bone resorption after extraction [10–13]. The immediate denture is used as a guide for tissue healing. Resorption is said to be more limited with, rather than without, a denture or with a transformed existing partial denture [10–13].

Yet a comparison of crestal bone resorption with and without immediate complete denture is not easily established. Few data on resorption in the maxilla after multiple extractions are available. The centripetal direction was demonstrated [14–17], but resorption severity is considered to increase when several teeth are removed [18]. However, the extent of resorption is highly variable, depending on factors such as patient profile and extraction conditions [14, 19]. Resorption for patients with edentulism seems to be more intense in the first 3–6 months [20–22]. In a 30-month follow-up study, Watt et al found a 52 % of resorption of the bone volume in the first 3 months after extraction and a 72 % resorption at the end of the first year [22].

Resorption was also measured in protocols including extractions and the immediate placement of maxillary complete dentures [10, 11, 23–26]. The mean resorption reported in those studies was 3.3 mm vertically and 2 mm in width after 1 year, at the expense of the buccal alveolar bone.

To our knowledge, only one study evaluated the interest of placing a bone substitute material in the alveolar sockets to reduce resorption after teeth extractions [23]. In 1973, Bergstedt et al. used xenografts treated with ethylenediamine and found reduced vertical and horizontal resorption of the ridge.

Different graft materials can be used for socket filling: from an osseous origin (autograft, allograft, or xenograft) or from alloplastic materials (apatites, calcium phosphate, bioactive glass, etc.). They can be used alone or combined with resorbable or nonresorbable membranes. Systematic reviews have shown that the filling sockets to preserve bone is of interest but have been unable to identify the best material or strategy [27–31].

The biomaterial chosen in the present protocol, Bio-Oss Collagen®, was recently investigated in human studies and showed good clinical results [32–38]. This xenograft material is composed of 90 % bovine cancellous bone mineral granules with the addition of 10 % purified porcine collagen. Araujo et al. published a protocol of filling single sockets in the anterior region [32]. Bone measurements were performed on 3D bucco-lingual reconstructions from cone beam computed tomography (CBCT). This technique appears to be the best tool for bone volume evaluation, with an accuracy and reliability of linear measurements equivalent to that of multislice computed tomography (MSCT) for use in the dental and maxillofacial region, with good radioprotection [39, 40].

The difficulty in cases of complete edentulism is in choosing a quantitative protocol to evaluate resorption. Previous studies used cephalometric tracings [10, 11, 23, 24, 26]. Michael et al. worked on casts [41]. These two approaches allow for repeated measurements at the different stages of healing. Cephalometry causes little deformation but only provides 2D images; it gives a general appreciation of resorption in the anterior area but only in the sagittal plane.

Reference points are crucial in providing a quantitative evaluation. With complete edentulism, no adjacent tooth can be used. Ridge references—such as the apical point of the socket [32]—were previously used, but they are questionable because crestal bone progressively remodels and resorbs. Moreover, distinguishing between cancellous bone, the biomaterial, and newly formed bone on radiographs or CBCT is debatable. Combining CBCT technology with reference points independent of the studied osseous site seems an innovative option in the peculiar situation of complete edentulism. The present study uses a radiographic index on a duplicate of the complete denture made of resin, with 20 % barium sulfate as reference points for measurements.

### Objective

The aim of this study is to evaluate the efficacy of a new therapeutic strategy for edentulism, associating maxillary immediate complete denture and bone grafting, compared with conventional maxillary immediate complete denture treatment without bone grafting in terms of bone volume preservation (height and width of the bone ridge).

The primary objective is to compare the bone ridge height 1 year after maxillary immediate complete denture placement with or without bone substitute material placed in incisivo-canine sockets.

The secondary objective is to compare bone ridge width 1 year after maxillary immediate complete denture placement with and without bone substitute material placed in incisivo-canine sockets.

### Hypothesis

The research hypothesis is that a new strategy associating maxillary immediate complete denture and bone substitute material is more effective in limiting ridge resorption than conventional immediate complete denture.

## Methods/design

### Study design

This trial is a single-center, randomized, single-blind, superiority trial with two balanced parallel arms. The trial received approval from the French Ethics Committee for the Protection of Persons (Comité de Protection de Personnes, trial number 13-019) in June 2013.

The Clinical Trial registration number is NCT02120053, and the trial was registered on 18 April 2014.

### Setting and location

The patients are being recruited from the dental consultation in Albert Chenevier-Henri Mondor Academic Hospital (Assistance Publique-Hôpitaux de Paris (AP-HP), France).

### Participants

#### Patient inclusion criteria

All patients requiring maxillary immediate complete denture are eligible for participation if they meet the inclusion criteria. Patients are included if they meet the following criteria:

- Are candidates for maxillary immediate complete denture, presenting a Kennedy Class I partial dentition (bilateral posterior tooth loss)
- Older than 18 years of age
- Have a healthy adherent gingiva
- Are willing to participate in the study and able to sign the consent form

During inclusion, the periodontal status and smoking habits of the patients are assessed. Because of the consequence of these two factors on bone healing, these factors are identified as prognostic factors and considered in the randomization.

**Fig. 1:**
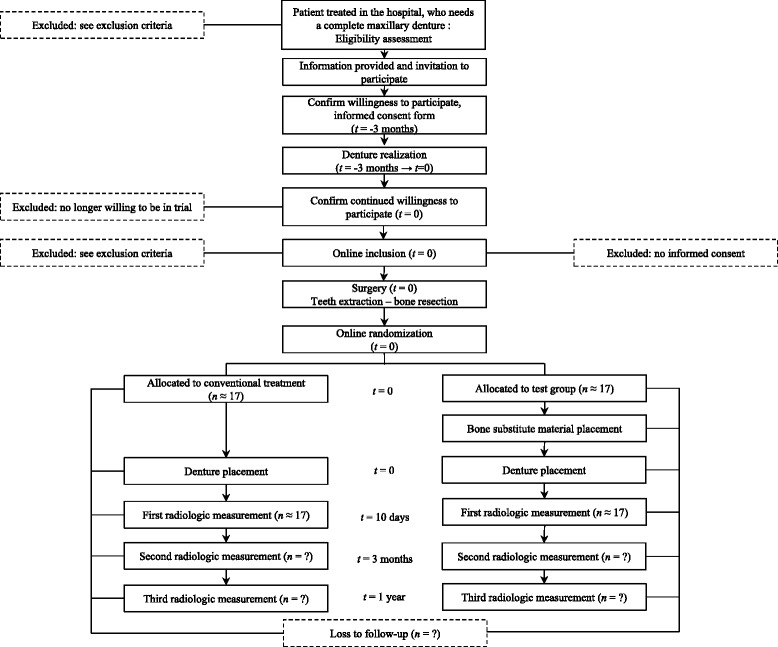
Participant flow diagram

Informed consent from each participant is obtained (Fig. 1).

### Patient exclusion criteria

Investigators meticulously screen the general health of the patients during the first interview. On inclusion day, the investigators check again for major medical conditions, using a questionnaire included in the case report form (CRF). Patients with any of the following conditions and attitudes are excluded:

- Medical conditions contraindicating oral surgery
- Progressive cancer
- History of radiotherapy in the head and neck region
- Major neurological disease
- Anticoagulant treatment with international normalized ratio < 2
- Valvulopathy, hematologic disease, or agranulocytosis
- Serious heart failure or recent myocardial infarction < 5 years
- Immune deficiency or AIDS
- Osteomalacia
- Hepatic or renal insufficiency
- Unregulated diabetes*
- Long-term steroidal treatment
- Bisphosphonate treatment
- Allergy to collagen
- Pregnant or nursing
- Staff specially protected
- Not affiliated with the social security system

* In case the patient is not sure about particular pathologies, a blood test is prescribed.

### Investigator inclusion criteria

Prosthodontic specialists and surgeons are required to be senior lecturers in the dental faculty and to be trained in complete denture realization. Three lecturers agreed to participate: one prosthodontist-surgeon (CRB), one surgeon (AH), and one prosthodontist (CW), so two investigator teams were created (team 1: CRB alone; team 2: AH and CW).

### Intervention

**Fig. 2:**
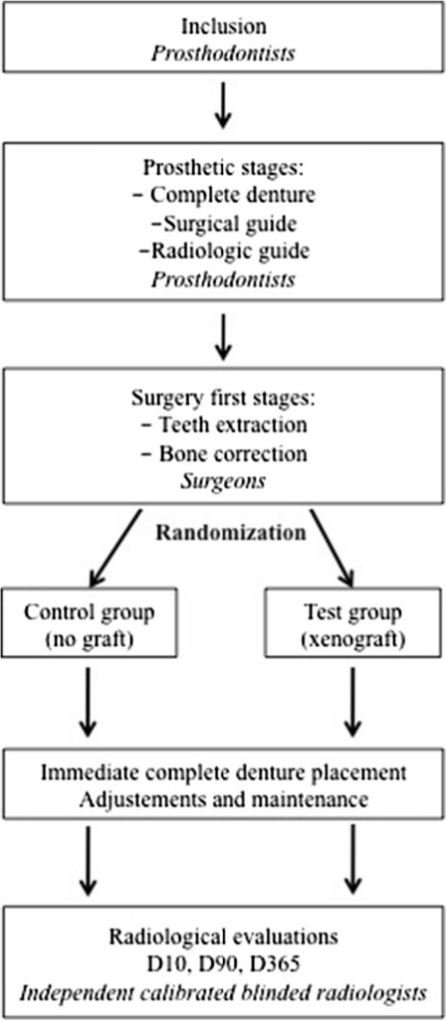
Study overview

At the maxilla, posterior teeth were previously extracted to obtain a Kennedy Class I partial dentition (bilateral posterior tooth loss). At least 3 months after the posterior teeth extraction, eligibility is assessed. Once eligibility is validated, the protocol is presented and explained to the patient by one of the investigators. Inclusion is validated after the patient signs consent (Fig. 1). Figure 2 gives an overview of the study.

#### Denture realization

The protocol follows the conventional procedure [3]:

- A preliminary alginate impression is made with a metal stock tray (rimlock) to obtain a cast on which a resin custom tray is realized.
- The final impression is obtained by using the resin custom tray. First, a compound border mold is made with Kerr compound (Kerr Dental, Orange, CA, USA) on the buccal vestibule of the edentulous area to register this part of the peripheral seal. The same material is used for the posterior palatal seal. Then, a heavy-bodied polyether material (Permadyne®, 3 M ESPE, Seefeld, Germany) is used in the labial vestibule to finalize the peripheral seal. The final impression involves use of light-bodied consistency polysulfide (Permlastic®, Kerr Dental, Orange, CA, USA). This impression is poured in stone according to the manufacturer’s instructions and involves a classical laboratory procedure to obtain the master cast.
- The vertical dimension of occlusion (VDO) is carefully assessed by using multiple methods such as evaluation of rest position, tactile sensitivity, and phonetics. Occlusion rims are made to record the maxillomandibular relationship. The height of the occlusion rim is modified to the VDO. The rims are prepared for centric relation (CR) registration. The maxillomandibular relationship is recorded at the correct VDO and CR. The occlusion rims are then placed on the master casts and transferred to an articulator.
- The artificial teeth are arranged to ensure cross-tooth, cross-arch conventional balanced occlusion for complete dentures. The practitioner and the patient approve the teeth arrangement during a try-in session.
- Before polymerization, the master cast is modified by the prosthodontist specialist to simulate dental extractions; the latter removes undercuts and anticipates bone resorption and tissue healing. The post dam is carved.
- Denture polymerization is achieved according to classical laboratory procedures.
- The denture is then finished and polished.

#### Surgical guide

A transparent resin surgical guide, which is a duplicate of the complete denture, is also polymerized.

#### Radiographic guide

**Fig. 3:**
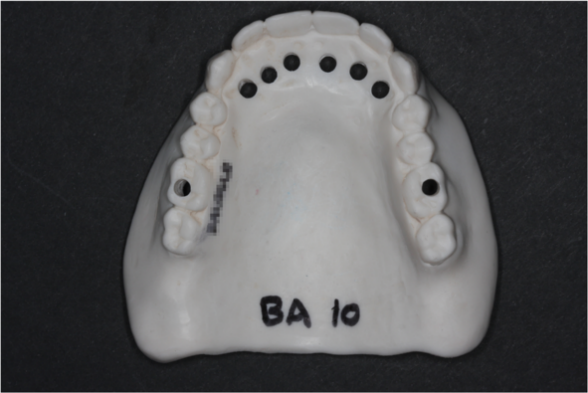
Radiographic guide with pits next to extraction sites

This is a second duplicate of the complete denture, made of acrylic resin containing barium sulfate powder (20 wt%), which provides radio opacity and is used as a radiographic guide for each patient (Fig. 3).

To locate the alveolar extraction sites around canines and incisors (fresh sockets) during the radiological examinations, 3-mm-wide pits are prepared in line with this radiological guide. Moreover, to facilitate the location of various cross-sections during the successive radiological examinations, two further pits next to 16 and 26 are also realized. The pits appear radiolucent in contrast to the radio-opaque duplicate on the reconstructions. They constitute start and stop reference points for numbering the bucco-lingual reconstructions during CBCT.

The duplicate of the patient denture provides an accurate site position index for reproducible measurements because of its intimate adaptation on the posterior ridge, palatal vault and mucosa, and maxillary suture [42]. The amount of the 20 wt% of the sulfate barium gives a readable contrast between the guide and the reference pits, with no artifact preventing bone contour visibility.

#### Surgery

Surgery proceeds as follows [3]:

- Patients undergo deep local anesthesia with Scandicaine 3 % (Septodont, St. Maur des Fosses, France) without vasoconstriction in the anterior portion of the upper jaw.
- A sulcular incision is made and extended with a distal wedge.
- Tooth removal is performed carefully to maintain the integrity of the labial and palatal bony plates.
- The wound is debrided carefully by curettage and the socket walls are inspected.
- A full-thickness gingival flap is lifted up to the mucogingival junction to enable bone correction. The surgical guide is then used to adjust the bone contour. Tissue whitening indicates compression areas where bone correction is necessary. Bone correction is performed until a homogeneous gingival whitening occurs under the guide and the post dam is well fitted.
- To prevent any bias during the final cast preparation and extraction protocol, randomization takes place only after extraction and immediately before eventual socket filling (Fig. 1).
- For patients allocated to the experimental group (receiving a graft): fresh sockets are filled with a bone substitute material (Bio-Oss Collagen®, Geistlich©, Wolhusen, Switzerland). Bio-Oss Collagen® is derived from the previous Bio-Oss® (bovine hydroxyapatite) with the addition of porcine fibrous collagen.
- No suture is needed for this procedure. Hemostasis control is obtained through compression with the immediate denture in place.
- After 10 min of hemostasis control with the guide, the denture is placed and the occlusion controlled. Painkillers and antibiotics are prescribed before surgery.

#### Post-operative

Post-operative procedures are as follows:

- The patient is instructed not to remove the immediate denture for 48 h.
- At D2, the prosthodontic specialist cautiously removes the denture and cleans it. In this way, the specialist controls the healing. The patient is shown how to replace and remove the denture.
- Maintenance appointments are scheduled at D4, D7, D15, and each week as long as necessary to ensure comfort with the new denture. During these appointments, mucosal healing is checked, and routine adjustments to the denture base and occlusion are performed.
- Baseline measurements of the crestal ridge are taken at D10 with CBCT. Intermediary and final measurements are scheduled at 3 and 12 months.

No special medical concomitant care or interventions are prohibited during the trial.

### Strategies to improve adherence to intervention protocols

Participants receive no financial compensation. In thanks for their participation, they are offered the treatment, the denture, and the radiological exams.

Patients are given a detailed document 1 week before the surgery to explain what they should do in the first week after the surgery; this includes instructions on the continuous wear of the denture for the first 2 days, hygiene, and nutritional advice.

Investigators all took part in the protocol conception so that they know it well.

### Outcomes

The primary outcome is to compare overall (the mean of all extraction sites) mean bone ridge height loss (D365–D10) at 1 year after maxillary immediate complete denture placement with or without bone substitution. A quantitative evaluation method using CBCT was designed.

The secondary outcomes are as follows:

- To compare mean bone ridge height loss, site by site (central incisor, lateral incisor, and canine), with the same technique after 3 months (D90-D10) and after 1 year (D365-D10)
- To compare mean bone ridge width, overall and site by site, with the same technique after 3 months (D90-D10) and after 1 year (D365-D10).

CBCT data collection and ridge measurements are performed in an independent specialized radiology clinic. For the study purposes, a CBCT unit (NewTom VGi QR s.r.l.©, Verona, Italy), with a 7.5 × 12-cm field, was selected. CBCT data are collected at D10, D90, and D365. The volume CT dose index ranges from to 2 to 4 mGy per examination, according to the patient morphology.

An original protocol was developed to perform reproducible measurements and to compare ridge resorption in the two treatment arms. Bone height and width are measured on CBCT reconstructions by using a radio-opaque denture duplicate specifically designed for the study purposes.

#### Radiographic analysis

In the native dicom file, the study volume is selected to be parallel with the palate (ENA-ENP). The panoramic section is then outlined by linking the center of the different pits (i.e., the incisivo-canine pits and those at 16 and 26). Bucco-lingual reconstructions 1-mm thick are spaced 1 mm apart, beginning at the start slot and ending at the stop slot. Radiolucent indices (pits) of the studied incisivo-canine sites serve as markers for the choice of the cross-section. Therefore, evaluators determine the examined cross-section for each extraction site, indexed according to its number. This cross-section becomes a reference, serving as the section to study for each evaluation of each site.

**Fig. 4:**
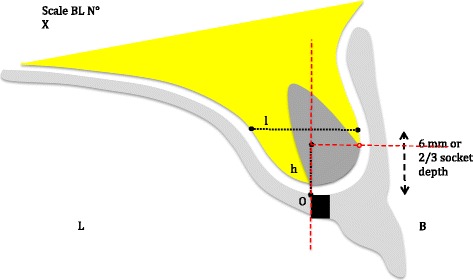
Schematic measurement principles. h is the height measurement, the distance between the palatal pit end (reference point O) and the buccal top of the outer cortical bone. l is the bone ridge width, measured on a bucco-lingual line crossing the alveolar socket, perpendicular to the palatal pit axis and at a distance of 6 mm or two thirds of the socket depth (depending on the patient’s bone morphology) from the reference point O

Two measurements are taken for each extraction site during the three evaluation times :

- A vertical measurement (h): the distance between the palatal pit end (reference point O) and the buccal top of the outer cortical bone.
- A horizontal measurement (l): the bone ridge width is measured on a bucco-lingual line crossing the alveolar socket, perpendicular to the palatal pit axis and at 6 mm or two thirds of the socket depth from the reference point O. Two thirds of the socket depth is only used when the ridge general height, palatal curvature, and periodontal resorption make the 6-mm index irrelevant. The choice of horizontal measurement index is made on the first radiological examination and used in the next evaluations (Fig. 4).

Two independent calibrated radiologists perform each measurement with blinding to the treatment arm. Inter-operator reproducibility is evaluated with an intraclass correlation coefficient (ICC) for each osseous height and width measurement.

### Participant timeline

**Fig. 5:**
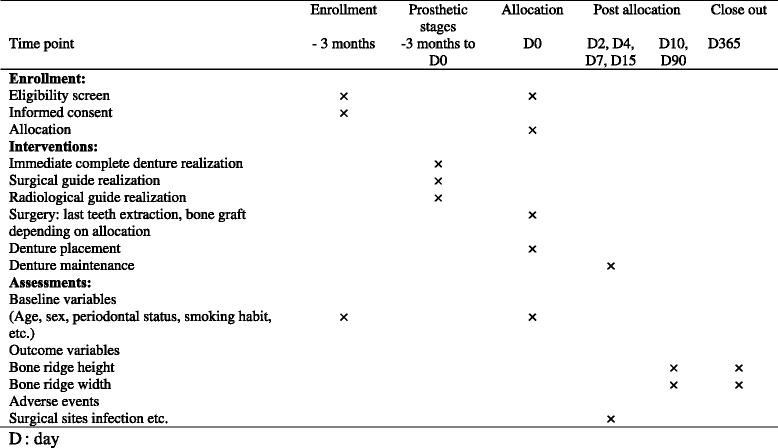
Participant’s timeline

Figure 5 illustrates the participant’s timeline.

### Sample size

The number of participants needed to achieve study objectives was estimated to be 34. From a literature review [10, 11, 24–26], we estimated that the average 1-year resorption would be 3 mm in the control group (SD = 1.5 mm) and that 2 mm of bone preservation in the test group would be clinically significant. With an alpha risk of 5 % and a beta risk of 10 %, 12 patients per group were required. Because two operator groups would be involved, extra variability could be expected. No ICC was reported in reports identified by our literature review, so ICC 0.02 was chosen (coefficient obtained in a previous clinical study conducted by our research unit [43]). Based on this ICC, the sample size was increased, and 29 patients (12 × 2 × (1 + (11 x 0.02))) were needed. We decided to include five extra patients in case some were lost to follow-up.

### Allocation

The biomaterials laboratory clinical research unit (URB2I-EA4462, Faculty of Dentistry, Paris Descartes University) is responsible for the randomization.

#### Sequence generation

Treatment allocation is attributed by minimization and takes into account the following main prognostic factors: operator (two teams: prosthodontics practitioner/surgeon), smoking habit (<10 cigarettes/day or ≥ 10 cigarettes/day), and periodontal disease evolution (< or ≥ half the radicular height). To reduce predictability, 30 % randomness is included into the minimization algorithm (this method was chosen to minimize imbalance and predictability, according to the Hermes simulation software [44]).

#### Allocation concealment mechanism and implementation

The patient has to be included/registered online with the RandoWeb® software (AP-HP, Paris, France) before complete denture delivery, so all data are recorded prospectively without inclusion being influenced by the allocation result.

During the surgery, just after extraction of the last teeth, the allocation result can be obtained online by the surgeon if the patient has been included/registered online beforehand. The treatment arm is then attributed (control group: conventional surgery without osseous filling; experimental group: alternative surgery with socket filling). Operators have access only to the last inclusion result in the randomization table, to limit predictability.

HFC conceived the minimization algorithm and Vincent Morice implemented it into the RandoWeb software.

### Blinding

The study is described as a double-blind trial (patients and outcome assessors). Surgeons and prosthodontists cannot be blinded, but radiologists perform measurements blindly. Operators do not communicate with the patient about the treatment to ensure independent radiologist measurements of ridge height and width.

### Data collection

Investigators will use a CRF to record all items required for the outcomes analysis. A clinical research assistant visits the investigation center every 6 months to monitor the collection of data (by checking that no CRF field is incomplete) and to assess the quality (by comparing the data in the medical record, the data entered through the online inclusion and randomization software RandoWeb (AP-HP, Paris; http://randoweb.aphp.fr), and the data in the CRF).

Two independent calibrated operators, with blinding to the treatment arm, perform radiologic measurements.

The data are transmitted to an adjudication committee to harmonize the collection standardization and data evaluation. This committee consists of a radiologist, a prosthodontist, and a surgeon. It focuses on the primary outcome.

Participant retention should not be difficult for the first two radiological exams because routine appointments for maintenance usually continue for up to 2 months, when a second CBCT appointment occurs. Patients are advised that denture maintenance will occur 1 year after placement at the time of the third radiologic exam. The clinical research assistant calls 1 month before the date of the third CBCT to make the appointment. In case of five unsuccessful calls, two registered letters with acknowledgement of receipt are sent.

### Data management

Data are recorded on the CRF by investigators and evaluators. All fields must be completed.

Patient data are anonymous because patients will be identified by their inclusion number (the first letter of their first and last name and date of birth only will be registered on the CRF).

For statistical analysis, the data are recorded in an Excel spreadsheet before being analyzed with Stata 12.

### Statistical methods

The data will be analyzed by an independent statistician (HFC, in collaboration with the Henri Mondor Hospital Clinical Research Unit). The unit of analysis will be the extraction site (a maximum of six teeth will be extracted per patient). The demographic and clinical characteristics of the patients, the alveolar bone, and the extracted teeth will be described for both treatment arms with the usual statistics: mean and SD or median and interquartile ranges for quantitative variables and number of subjects and percentages for the qualitative variables [53]. The analyses will be performed according to the intent-to-treat principle.

#### Primary outcome analysis

The main analysis will compare the final loss (D365-D10) of bone ridge height between the experimental and control group. This main analysis will be adjusted on the following prespecified variables: operator team, smoking habit, periodontal disease evolution, and age and sex of the patient. A linear mixed model (probably marginal) will be used to account for the correlation between the different bone ridge height values in the same patient (level 1 will be the extraction site, and level 2 will be the patient; level 3 will be the investigator team, if necessary). The main analysis will take into account missing outcome data by multiple imputation, with the assumption that data are missing at random. We will report the unadjusted analysis as well; mean final loss of bone ridge height will be compared between the experimental and control group by Mann-Whitney test. All *p* values will be two-tailed, with a significance level of 0.05.

#### Secondary outcomes analyses

The same analyses will be used to compare final loss (D365-D10) of bone ridge width, final loss of bone ridge height and width at the different extraction sites (central incisor, lateral incisor and canine) and intermediate loss (D90-D10) of bone ridge height and width.

A repeated data model will be used to compare bone loss speed between the experimental and control group.

#### Subgroup analyses

We will perform subgroup analyses of the following variables: smoking habit, periodontal disease evolution (clinical attachment level, bleeding on probing, probing pocket depth, and periodontal biotype), age and sex of the patient, antagonist (teeth/removable partial denture/removable complete denture), and maxillary posterior alveolar ridge resorption. The testing interaction in nine independent subgroups implies a 37 % risk of finding at least one false positive.

### Data monitoring, harms and auditing

The data will be monitored by an independent clinical research assistant who will compare the data entered in the CRF with those in the patient’s clinical record. In case of disagreement, the patient’s operator/investigator will be asked to clarify the data. No interim analysis is planned. Concerning harms monitoring, the CRF contains two adverse events forms: one concerning general health and one that is treatment-related.

Trial management may be audited by the French Department of Health at any time; the audit would be independent of investigators and the sponsor. Investigators will not have access to the final trial data set; the latter will be accessed by clinical research assistants, data managers, and statisticians only.

### Ethical considerations

**Fig. 6:**
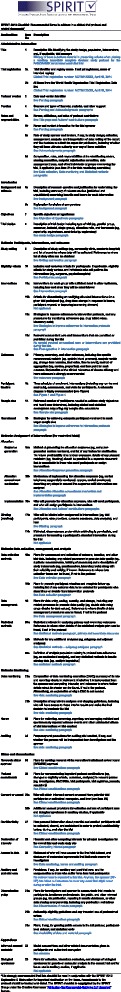
SPIRIT 2013 checklist

The trial received approval from the French Ethics Committee for the Protection of Persons (Comité de Protection de Personnes, trial number 13-019) in June 2013. The protocol is registered with the Agence Nationale pour la Sécurité du médicament et des Produits de Santé (ANSM, French National Agency for Medicines and Health Products Safety (2013-A00440-45 (IDRCB/Eudract)) and ClinicalTrials.gov (no. NCT02120053, 18 April 2014). All amendments to the protocol will be justified, submitted to the scientific board, accepted by the CPP, and recorded by the ANSM. Changes and amendments will be also recorded at ClinicalTrials.gov. Informed consent will be obtained from trial participants after the trial is explained by an investigator or operator of the corresponding center. Patients are informed that they have the right to withdraw from the study at any time without giving reasons. Regardless of withdrawal, patients will be provided any treatment in their best interest. Withdrawal will be documented. Data confidentiality was audited by the Comité National Informatique et Liberté (National Committee of Informatics and Freedom); last and first names of included patients are not recorded in the database. Moreover, the authors followed the SPIRIT 2013 checklist (Fig. 6).

### Dissemination of results

The Consolidated Standards of Reporting Trials (CONSORT) guidelines will be used to report the results of this study, and the results will be published in international peer-reviewed journals [45]. Authors of the publications will be people involved in the elaboration of the protocol, the implementation and conduct of the trial, and the writing of the manuscript and report. The results related to the main objective will be authored by the coordinator, the methodologists, the investigators, and other people who will have contributed significantly to the planning of the trial, its implementation, or the writing of the report.

A summary of the study results will be posted at ClinicalTrials.gov to allow general access to the findings.

Data sharing will be at the participant level. Access to the full protocol can be granted to anyone upon request.

## Discussion

Bone preservation is a current issue, in terms of the tooth-extraction strategy and especially the use of bone-substitute material. Authors focus mostly on single-tooth extraction, and no recent study has examined resorption after multiple extractions. From older studies [10, 11, 23–26], the centripetal direction of resorption in the maxillary anterior region is known, as is its extent in both the vertical and horizontal direction. Only Bergstedt et al. investigated the use of bone-substitute material after multiple extractions and placement of an immediate complete denture. The authors showed reduced resorption when using ethylenediamine-treated bone (vertical resorption 3.92 mm without socket filling versus 2.73 mm with filling; horizontal resorption 2.24 mm without socket filling versus 1.81 mm with filling). This reduction was statistically significant if teeth were in good periodontal condition before extraction, determined by an alveolar resorption less than half the root length. Tooth decay and periodontitis are the two main indications for tooth extraction. This result was considered in the present study protocol, and periodontal condition was retained as a randomization parameter. The two other randomization parameters are the prosthodontist/surgeon team and smoking habit. The adverse effect of tobacco on inflammation and healing are well demonstrated [46, 47].

Evaluation times were limited to three times: 10 days, 3 months, and 1 year after extraction [48, 49]. As underlined by Morjaria et al., most studies use bone reference measurements performed before extractions [27]. Actually, the biomaterial is present on the CBCT images after surgery and may affect radiologist blinding to treatment [27]. However, we did not use this procedure in this study. Indeed, the ridge has to be corrected at the end of the surgery to allow for denture insertion. In these conditions, ridge reference measurements can only be performed after extractions. After 10 days, the edema is resorbed, and the radiographic guide can be inserted without any pain or misfitting of the guide. The clot is already well constituted, but osseous remodeling is only beginning and is radiologically undetectable.

The major advantage of this innovative clinical trial lies in its methodological approach (randomized controlled trial) and the relevance of the measurement protocol of primary and secondary outcomes.

The results of this study may show the benefits of bone substitute materials in the immediate complete denture technique in order to limit bone resorption in the maxillary anterior region. This would increase the retention and stability of the denture and optimize an implant treatment subsequently.

## Trial status

Recruiting

## Abbreviations

ANSM: Agence Nationale pour la Sécurité du Médicament et des Produits de Santé (French national agency for medicines and health products safety)
AP-HP: Assistance Publique – Hopitaux de Paris (group of public hospitals in Paris)
CBCT: Cone-beam computed tomography
CONSORT: Consolidated Standards of Reporting Trials
CPP: Comité de Protection de Personnes (French Ethics Committee for the Protection of Persons)
CRF: Case report form
ICC: Intraclass correlation coefficient
MSCT: Multislice computed tomography
VDO: Vertical dimension of occlusion
